# Familial adenomatous polyposis

**DOI:** 10.1186/1750-1172-4-22

**Published:** 2009-10-12

**Authors:** Elizabeth Half, Dani Bercovich, Paul Rozen

**Affiliations:** 1 Familial Cancer Clinic, Gastroenterology Dept, Meir Hospital, Kfar Saba, Israel; 2 Human Molecular Genetics & Pharmacogenetics, Migal - Galilee Bio-Technology Center, Kiryat-Shmona, 11016, Israel; 3 Tel-Hai Academic College, Israel; 4 Sestopali Fund for Gastrointestinal Cancer Prevention, Dept of Gastroenterology, Tel Aviv Medical Center, 6 Weizmann St, Tel Aviv, 64239, Israel; 5 Tel Aviv University Medical School, Israel

## Abstract

Familial adenomatous polyposis (FAP) is characterized by the development of many tens to thousands of adenomas in the rectum and colon during the second decade of life. FAP has an incidence at birth of about 1/8,300, it manifests equally in both sexes, and accounts for less than 1% of colorectal cancer (CRC) cases. In the European Union, prevalence has been estimated at 1/11,300-37,600. Most patients are asymptomatic for years until the adenomas are large and numerous, and cause rectal bleeding or even anemia, or cancer develops. Generally, cancers start to develop a decade after the appearance of the polyps. Nonspecific symptoms may include constipation or diarrhea, abdominal pain, palpable abdominal masses and weight loss. FAP may present with some extraintestinal manifestations such as osteomas, dental abnormalities (unerupted teeth, congenital absence of one or more teeth, supernumerary teeth, dentigerous cysts and odontomas), congenital hypertrophy of the retinal pigment epithelium (CHRPE), desmoid tumors, and extracolonic cancers (thyroid, liver, bile ducts and central nervous system). A less aggressive variant of FAP, attenuated FAP (AFAP), is characterized by fewer colorectal adenomatous polyps (usually 10 to 100), later age of adenoma appearance and a lower cancer risk. Some lesions (skull and mandible osteomas, dental abnormalities, and fibromas on the scalp, shoulders, arms and back) are indicative of the Gardner variant of FAP. Classic FAP is inherited in an autosomal dominant manner and results from a germline mutation in the adenomatous polyposis (*APC*) gene. Most patients (~70%) have a family history of colorectal polyps and cancer. In a subset of individuals, a *MUTYH *mutation causes a recessively inherited polyposis condition, *MUTYH*-associated polyposis (MAP), which is characterized by a slightly increased risk of developing CRC and polyps/adenomas in both the upper and lower gastrointestinal tract. Diagnosis is based on a suggestive family history, clinical findings, and large bowel endoscopy or full colonoscopy. Whenever possible, the clinical diagnosis should be confirmed by genetic testing. When the *APC *mutation in the family has been identified, genetic testing of all first-degree relatives should be performed. Presymptomatic and prenatal (amniocentesis and chorionic villous sampling), and even preimplantation genetic testing is possible. Referral to a geneticist or genetic counselor is mandatory. Differential diagnoses include other disorders causing multiple polyps (such as Peutz-Jeghers syndrome, familial juvenile polyps or hyperplastic polyposis, hereditary mixed polyposis syndromes, and Lynch syndrome). Cancer prevention and maintaining a good quality of life are the main goals of management and regular and systematic follow-up and supportive care should be offered to all patients. By the late teens or early twenties, colorectal cancer prophylactic surgery is advocated. The recommended alternatives are total proctocolectomy and ileoanal pouch or ileorectal anastomosis for AFAP. Duodenal cancer and desmoids are the two main causes of mortality after total colectomy, they need to be identified early and treated. Upper endoscopy is necessary for surveillance to reduce the risk of ampullary and duodenal cancer. Patients with progressive tumors and unresectable disease may respond or stabilize with a combination of cytotoxic chemotherapy and surgery (when possible to perform). Adjunctive therapy with celecoxib has been approved by the US Food and Drug Administration and the European Medicines Agency in patients with FAP. Individuals with FAP carry a 100% risk of CRC; however, this risk is reduced significantly when patients enter a screening-treatment program.

## Disease names

Familial adenomatous polyposis (FAP)

## Synonyms

Familial polyposis coli

## Variants or FAP-associated conditions

Attenuated FAP is a milder form of FAP

Gardner's syndrome is a clinical variant of FAP where the extra-colonic features are prominent

Turcot syndrome refers to FAP and having a medulloblastoma brain tumor

## Definition

FAP is an autosomal dominant disease that is classically characterized by the development of hundreds to thousands of adenomas in the rectum and colon during the second decade of life. Almost all patients will develop colorectal cancer (CRC) if they are not identified and treated at an early stage. However, today it is unusual for patients to present with CRC as the majority of patients are diagnosed before cancer develops.

Attenuated FAP is a milder form that is characterized by fewer adenomas, a later age of adenoma development and cancer diagnosis.

## Epidemiology

**Figure 1:**
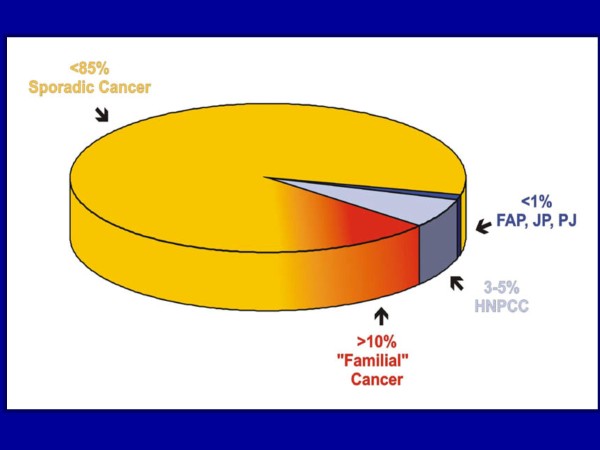
**Relative and approximate contributions of familial causes to the incidence of colorectal cancer**. FAP - Familial adenomatous polyposis, JP - Familial juvenile polyposis, PJ - Peutz-Jeghers syndrome, HNPCC - Hereditary nonpolyposis colorectal cancer (Lynch Syndrome). Note the very small contribution of FAP to the etiology of cancer.

Worldwide, CRC is a major cause of cancer associated morbidity and mortality. Its incidence varies considerably among different populations with the highest incidence reported from Western and industrialized countries. Worldwide, about 85% of CRCs are considered to be sporadic, while approximately 15% are familial with FAP accounting for less than 1% (Fig. 1). However, FAP is one of the best known and understood genetic diseases.

In many countries there are local FAP registries; however it is difficult to obtain accurate nation-wide data. In the UK, Reed and Neel presented a detailed genetic study in 1955 and calculated the incidence of FAP at birth to be 1:8,300 [1]. In 1975, Alm presented an incidence rate of 1:7,645 in Sweden [2]. These estimates were based on clinical criteria before the availability of mutation analysis and recognition of all the clinical variants and differential diagnoses. In 2009, the European Medicines Agency (EMEA) estimated that FAP affected approximately 3-10/100,000 people in the European Union which is equivalent to 11,300 - 37,600 individuals [3]. Clinically, FAP manifests equally in both sexes by the late teens and in the twenties age group.

## Clinical description

Symptoms are uncommon in the child and adolescent until the adenomas are large and numerous so as to cause rectal bleeding or even anemia. Other non-specific complaints such as change in bowel habits, constipation, or diarrhea, abdominal pains or palpable abdominal masses or weight loss in young patients can lead to recto-sigmoid examination and identification of polyps suggestive of FAP. FAP can present with extraintestinal manifestations such as osteomas, dental abnormalities (unerupted teeth, congenital absence of one or more teeth, supernumerary teeth, dentigerous cysts, and odontomas), congenital hypertrophy of the retinal pigment epithelium (CHRPE), desmoid tumors, or extracolonic cancers (thyroid, liver, bile ducts, central nervous system), (see below). Some lesions (skull and mandible osteomas, dental abnormalities, fibromas on the scalp, shoulders, arms, and back) are indicative of the Gardner variant of FAP. Today the condition should rarely present as a colonic or even as an extra-colonic malignancy.

### Colonic manifestations

**Figure 2:**
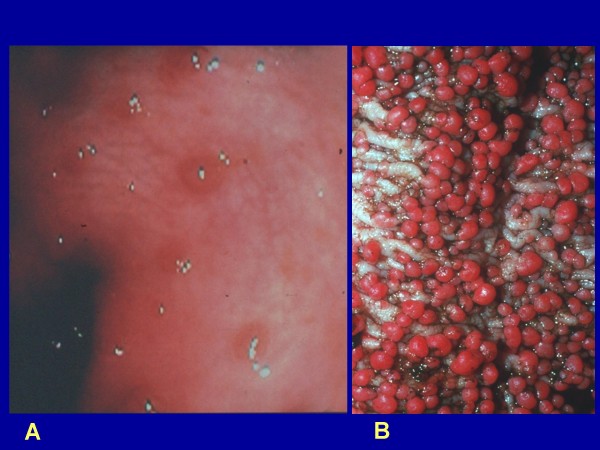
**Panel A shows the endoscopic appearance of early FAP adenomas; panel B shows the endoscopic appearance of established, multiple, FAP adenomas**.

Classic FAP is characterized by the presence of hundreds to thousands of colorectal adenomas of different sizes. Today this is rarely seen in countries with well developed public health services. In the majority of patients polyps begin to develop during childhood, mostly in the distal colon (rectosigmoid) as small intramucosal nodules (Fig. 2A). By the time of adolescence, the polyps are usually identified throughout the colon and, thereafter, increase in size and numbers (Fig. 2B). About half of FAP patients develop adenomas by 15 years of age and 95% by age 35 years [4].

Generally, cancers start to develop a decade after the appearance of the polyps. So, if the colon is left intact, the majority of patients with FAP eventually develop CRC by the ages 40-50 years. However, it should be emphasized that, although uncommon, CRC can develop in children or in older adults.

### Other gastrointestinal manifestations

Individuals with FAP can also develop a variety of extra-colonic gastrointestinal manifestations (3A).

**Figure 3:**
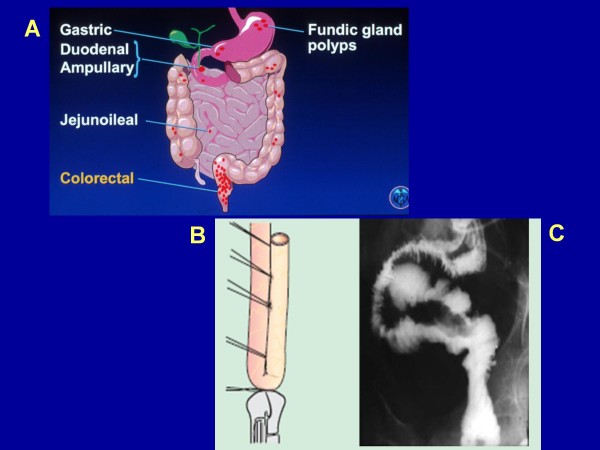
**Panel A illustrates the extracolonic upper gastrointestinal polyp manifestations of FAP (Used with permission and adapted from the American Gastroenterology Association Institute's Clinical Teaching Unit 11, copyright)**. Fig. B is a schematic illustration of a "J-pouch", formed from the distal small intestine and attached to the anal sphincter. The shorter limb of the "J" acting as a fecal reservoir; Fig. C is an x-ray demonstration of this pouch (Figures provided by Dr. A. Deutsch, Petach Tikva and reproduced, with permission of the publishers, from reference [70]: Rozen P, Levin B, Young GP: Who are at risk for familial colorectal cancer and how can they be managed? In *Colorectal Cancer in Clinical Practice: Prevention, Early Detection and Management *Edited by: Rozen P, Young GP, Levin B, Spann SJ. London, Ed 2, Taylor and Francis 2002:55-66).

In the stomach, *fundic gland polyps *(FGP) develop in 90% of patients with FAP. They are of special interest since, in contrast to the benign nature of sporadic FGP, 40% of these lesions in individuals with FAP have been shown to have adenomatous features, but rarely do progress to cancer [5]. FGPs in FAP patients are pathogenetically distinct from sporadic FGPs. Somatic, second-hit *APC *gene alterations, which precede morphological dysplasia in many FAP-associated FGPs, indicate that FGPs arising in the setting of FAP are neoplastic lesions [6].

*Adenomatous polyps in the duodenum *(mainly in the 2^nd ^and 3^rd ^parts) and *periampullary region*. In one series they developed in approximately 90% of individuals with FAP, 10-20 years after diagnosis of colorectal polyps [7]. The lifetime risk of duodenal adenomas has been reported to reach 100% [8,9]. Spigelman's classification of duodenal polyps is a scale, based on polyp number, size, histology, and severity of dysplasia (Table 1). It is estimated that about 5% of duodenal, and specifically periampulary polyps, progress to cancer within 10 years [10]. While rare in the general population, the risk of duodenal or periampullary cancer is increased several hundred fold in FAP patients [7]. Duodenal polyposis usually progresses in an orderly fashion through increasing Spigelman stage [7], but cancer can present in patients under surveillance with lower Spigelman stages being identified in their penultimate examinations [10-12]. Pancreatitis can be the result of ampullary adenoma or as a presentation of malignancy.

**Table 1:** Spigelman classification for duodenal polyposis in FAP

	**Stage 1**	**Stage 2**	**Stage 3**
Polyp number	1-4	5-20	>20
Polyp size (mm)	1-4	5-10	>10
Histology	Tubular	Tubulovillous	Villous
Dysplasia	Low grade	Low grade	High grade

Stage 0, 0 points; stage I, 1-4 points; stage II, 5-6 points; stage III, 7-8 points; stage IV, 9-12 points. Points are accumulated for polyps' number, size, histology and severity of dysplasia. Stage I (1-4 points) indicates mild disease, whereas stage III-IV (7-12 points) implies severe duodenal polyposis. Derived from reference [7].

*Small bowel adenomas*. It is well known that individuals with FAP carry a risk of small bowel polyps and even cancer although at a much lower rate than duodenal and ampulary neoplasms. The exact incidence of small bowel polyps is unknown and is generally dependent on the examining methodology used for evaluation. Bertoni G *et al.*, in 1993, studied 16 patients with FAP by push enteroscopy and detected jejunal polyps in 50% of subjects [13]. Later, with the use of double balloon enteroscopy or capsule endoscopy, the rate of jejunal and ileal polyps was estimated to be between 30-75% [14-16]. The rate of small bowel malignancy is much lower than ampullary or duodenal cancer. However, the treating physician should be aware of this possibility and be ready to implement surveillance, which will be discussed.

### Extra-intestinal manifestations

**Figure 4:**
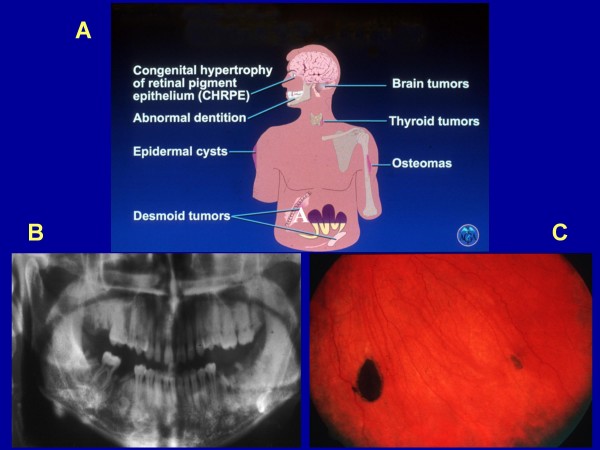
**Panel A illustrates the extraintestinal manifestations of FAP (Used with permission and adapted from the American Gastroenterology Association Institute's Clinical Teaching Unit 11, copyright)**. Panel B is a panoramic jaw X-ray showing mandibular areas of osteoslerosis that can be found in some FAP patients but is not diagnostic of FAP. Panel C shows retinal pigmentation (CHRPE) that can be found in some FAP patients but is not diagnostic of FAP.

Extraintestinal manifestations of FAP, which are rarely malignant, include: *cutaneous lesions *such as *fibromas, lipomas, sebaceous *and *epidermoid cysts*, *and nasopharyngeal angiofibromas *(Fig 4A). Osteomas can be palpated or seen, they can be identified as occult radio-opaque jaw lesions, and dental abnormalities which can be disfiguring (Fig. 4B). Gardner described the presence of these extraintestinal lesions and this phenotypic variant is named after him as "Gardner syndrome" [17]. *Congenital hypertrophy of the retinal pigment epithelium *(CHRPE) is a patch(s) of discoloration in the ocular fundus but is not specific for FAP (Fig. 4C). Almost all patients with CHRPE do not have symptoms and the lesions are found during examination of the dilated eye. However, when multiple bilateral lesions occur it can be a sensitive phenotypic marker and calls for screening. Low-grade adenocarcinoma has been described in these lesions [18,19].

Individuals with FAP may develop soft-tissue tumors (*desmoid tumors*) in the mesentery, abdominal wall or areas of scars. These tumors are considered benign, but by progressive enlargement and consequently pressure on gastrointestinal or urinary tracts, local nervous or vascular system, can be life threatening. Desmoids can be both a cause of severe morbidity as well as mortality. Over a lifetime, desmoid tumors occur in approximately 8% of men and 13% of women with FAP [20].

#### Other extracolonic malignancies

Other extra-colonic malignancies that are associated with FAP include *pancreatic mucinous adenocarcinomas, liver (hepatoblastoma)*, and *brain tumors*. In 1959, Turcot and colleagues described two teenaged siblings with adenomatous polyps of the colorectum in whom malignant tumors of the central nervous system developed [21]. Later it was noticed that "Turcot syndrome" is heterogeneous, encompassing at least two subtypes. The first is characterized by a germline mutation in one of the DNA mismatch repair genes, such as *hPSM2 *or *hMLH1 and *presents with the occurrence of glioblastoma in patients with Lynch syndrome (hereditary non-polyposis colon cancer, HNPCC). A second Turcot subtype is characterized by medulloblastoma, or rarely glioblastoma, in the setting of FAP and *APC *germline mutations [22]. *Thyroid cancer *in a FAP patient was first described in 1949 by Crail. Young women (less than 35 years of age) are at particular high risk of developing thyroid cancer, at a rate of approximately 160 times that of the general population [23,24].

### Attenuated familial adenomatous polyposis (AFAP)

**Figure 5:**
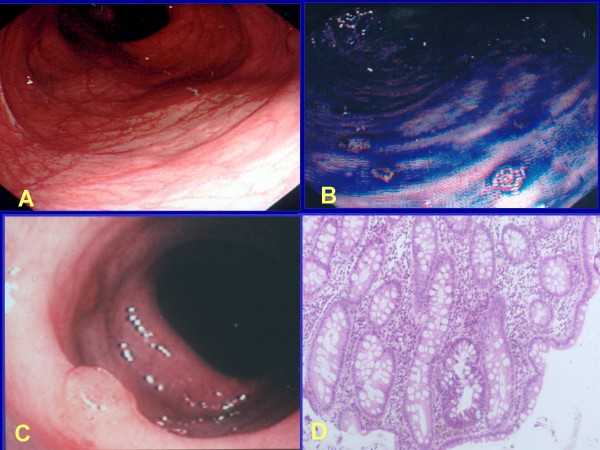
**Panel A shows the endoscopic appearance of early FAP and the difficulty in identifying adenomas; Panel B shows the endoscopic appearance after spraying with dilute Indian ink contrast chromoendoscopy; note the easier identification of small polyps (Figures reproduced from reference **[85]: **P Rozen, F Macrae, Familial adenomatous polyposis: The practical applications of clinical and molecular screening. Familial Cancer, 2006; 5:227-335, with kind permission of Springer Science and Business Media)**. Panel C shows the occasional adenoma seen in attenuated FAP and the difficulty in making the endoscopic diagnosis of FAP. Panel D demonstrates the usefulness of taking random biopsies in attenuated FAP and finding an intramucosal microadenoma, consistent with the diagnosis of FAP. Note the appearance of dark stained and elongated dysplastic nuclei in a distorted crypt that does not protrude above the surrounding normal surface epithelium (Figures reproduced, with permission of the publishers, from reference [70]: Rozen P, Levin B, Young GP: Who are at risk for familial colorectal cancer and how can they be managed? In *Colorectal Cancer in Clinical Practice: Prevention, Early Detection and Management *Edited by: Rozen P, Young GP, Levin B, Spann SJ. London, Ed 2, Taylor and Francis 2002:55-66.

This is a less aggressive variant of FAP that is characterized by fewer colorectal adenomatous polyps (usually 10 to 100), later age of adenoma appearance (mean age of polyp diagnosis is 44 years) and cancer (mean age 56 years). Clinically, it can be confusing as often there is mainly proximal colonic involvement with polyps, and infrequent rectal involvement. Thus, it can be misdiagnosed as occurring in a patient with sporadic adenomas (Figs. 5A-C) however, it can also occur in families with members having full clinical features of FAP.

Although these individuals have a smaller polyp burden relative to classic FAP, they still have an increased risk of cancer that, in general, occurs approximately 10-15 years later. As in FAP the most prominent extracolonic findings are upper gastrointestinal polyps specifically, duodenal and gastric adenomas and fundic gland polyps. Gastric and breast adenocarcinomas, as well as hepatoblastoma, have also been documented in AFAP. Other extracolonic manifestations of FAP are rare [25].

## Etiopathogenesis

FAP is a genetic disorder resulting from a mutation in the adenomatous polyposis gene (*APC*) gene.

Most FAP patients have a family history of colorectal polyps and cancer, however, 25-30% of them are "*de novo"*, without clinical or genetic evidence of FAP in family members [26,27]. It is now recognized that this can be partially explained by being the result of germline mosaicism [28]. Classic FAP is inherited as an autosomal dominant trait and results from a germline *APC *mutation; AFAP is mostly caused by specific *APC *mutations. A subset of individuals with clinical features of FAP will instead carry a mutation in the *MUTYH *gene (to be discussed later).

## The APC gene in brief

*APC *is a tumor suppressor gene located on the long arm of chromosome 5 in band q21 (5q21). The coding region is divided into 15 exons and encodes a large protein (309 kilo-Daltons) [29]. The *APC *protein has multiple domains that mediate oligomerization as well as binding to a variety of intracellular proteins, which have an important role in cell adhesion, signal transduction and transcriptional activation.

### Normal APC structure and functions

The *APC *gene spans a region of 108,353bp (NC_000005). The mRNA is 10,719 bp long (NCBI# MN_000038) and has 16 exons. The mRNA codes for a protein of 2,843 amino acids long with a molecular weight of 310 kDa (NCBI# NP_000029). Most of the amino acids are in the last exon (exon-16 that is 8,689 bp long with 6,574 bp coding sequences). Only exons 2-15 are coding exons and have 653 amino acids and exon 16 has 2,190.

**Figure 6:**
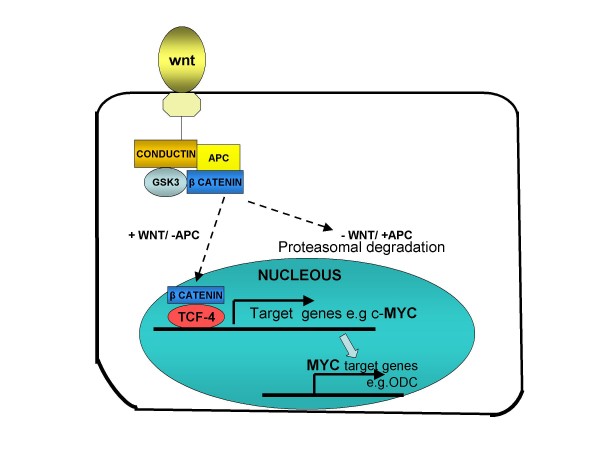
**Relationship between Wnt signaling and the APC tumor-suppressor gene in activating the β-catenin to enter the nucleus (no APC activity) for promoting genes expression and polyamine metabolism (ODC) in the colonic epithelium; or (with APC activity) for the proteasomal degradation in the colonic epithelium**.

*APC *is a classical tumor suppressor protein that plays a central role in Wnt signaling, in part by regulating the degradation of β-catenin. Wnt signals influence the stability of a protein complex containing β-catenin, conductin and GSK3 (glycogen synthase kinase 3). In the absence of Wnt or the presence of wild-type *APC *protein, β-catenin is degraded. In the presence of Wnt, or the absence of *APC *(as occurs in many colon cancers), β-catenin target genes including c-*myc *are expressed. *Myc *expression, in turn, leads to the expression of the polyamine ornithine decarboxylase (ODC) which is a proto-oncogene (Fig. 6). The *APC *gene product indirectly regulates transcription of a number of critical cell-proliferation genes, through its interaction with the transcription factor β-catenin. *APC *binding to β-catenin leads to ubiquitin-mediated β-catenin destruction; loss of *APC *function increases transcription of β-catenin targets. Homozygous *APC *truncation has been shown to affect chromosome attachment in cultured cells. Roles for *APC *in cell migration have been demonstrated *in-vitro *and in mouse models.

#### Disturbed APC structure and functions in brief

More than 300 different types of mutations are recognized today as the cause of FAP. Most of these mutations (insertions, deletions, nonsense mutations, *etc.*), result in a truncated protein. The most common mutation, occurring in about 10% of FAP patients, is a deletion mutation in codon 1309, the next most common, occurring in 5% of the patients, is a deletion at codon 1061.

#### APC disturbed structure and function in FAP in humans and animal models

Loss of normal *APC *function is known to be an early event in both familial and sporadic colon cancer pathogenesis, occurring at the pre-adenoma stage [30]. Generally, colon cancers show either chromosomal instability (CIN), which correlates with loss of *APC *function, or microsatellite instability, which correlates with loss of mismatch repair function, but not both. The genetically manipulated mouse model provides an excellent *in-vivo *system of human diseases and an opportunity to test therapies. The mouse model of FAP contains a point mutation in the *APC *gene; it develops numerous adenomas and was the first model used to study the involvement of the *APC *gene in intestinal tumorigenesis. The model has provided examples of modifying loci in mice and demonstrating the principle of genetic modulation of disease severity [31].

**Figure 7:**
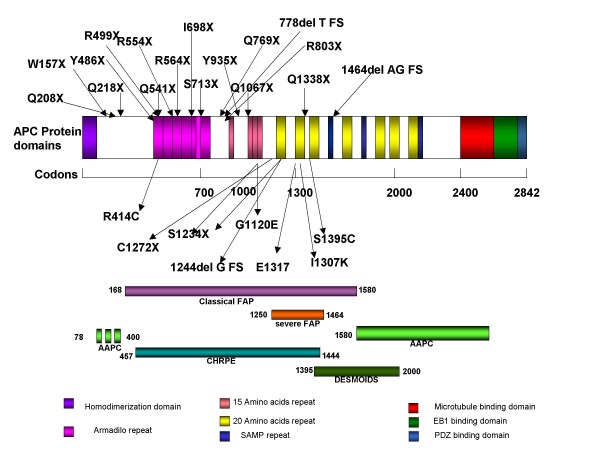
***APC *protein functional domains, several of the common mutations and their locations, in relation to the different FAP phenotypes**.

FAP is caused by a highly heterogeneous spectrum of point mutations and this represents a problem for molecular genetic diagnosis; all the mutations are chain terminating. Mutations typically cluster in, or just distal, to the armadillo repeat region and truncate near the middle of the protein [30,32] (Fig. 7). It is not known which is pathophysiologic - absence of the full-length protein or presence of the truncated version; evidence exists for both.

Mutational analysis of the *APC *gene indicates that the majority of germline mutations found in patients with FAP are nonsense mutations, leading to the formation of a truncated protein. More than 60% of *APC *mutations are found in the central region (between codons 1284 and 1580) of the protein, which is called the mutation cluster region (MCR) [33]. The MCR region coincides with a region in the *APC *gene that is important for the down-regulation of β-catenin, which suggests that this function is important for the pathogenesis of CRC. Subsequent studies demonstrated that *APC *and β-catenin are important parts of the Wnt signaling pathway (Fig. 6). The 5' coding region of exon 15 includes a second mutation-cluster region. In addition, there are several recurrently described mutations, of which two, at codon positions 1309 and 1061, account for as much as 30% of the germline *APC *mutations. The vast majority of mutations found in the *APC *gene represent truncating mutations (small deletions, 46%; small insertions, 10%; nonsense mutations, 28%). However, missense mutations (3%) and gross alterations (13%) have also been reported [34]. Recent data suggest that gross alterations in the *APC *gene may contribute more to this disease than previously reported; perhaps as many as 20% of FAP families may have a gross alteration.

#### Genotype-phenotype correlations in brief

Some correlation does exist between the sites of specific genetic mutations and the clinical manifestations of the disease, however, this correlation is not exact and differences do occur (to be discussed later) [35,36] (Fig. 7). Mutations contributing to classical FAP occur between exon 5 and the 5' portion of exon 15, whereas those associated with AFAP tend to cluster in the extreme 5' portion of the gene and the 3' portion of exon 15 proximal to codon 1517 or distal to codon 1900 [35,36]. Mutations between codons 1250 - 1464 are associated with profuse polyposis. Mutations at specific positions can cause CHRPE [37]; it is almost always absent if the protein-truncating mutation in the *APC *gene occurs before exon 9, but is consistently present if it occurs after this exon. Patients with a mutation between codons 1445 - 1578 do not express CHRPE, but can develop severe desmoid tumors [38].

## Diagnosis

The diagnosis of classic FAP is based on a suggestive family history and clinical findings. Whenever possible, the clinical diagnosis should be confirmed by genetic testing.

### Clinical diagnosis

The clinical diagnosis is dependent on the physician's suspicion and awareness. The patient may be completely asymptomatic and obtaining a detailed **family cancer history **is essential for a correct diagnosis, since in most cases some grandparents, parents and siblings will be affected. Asking simple questions like "has anyone in your family had cancer? Which cancer? And at what age?" is important information. Alternatively, rectal bleeding or abdominal complaints may develop depending on the stage of disease *i.e.*, polyp burden or stage of cancer. For the astute physician, identification of extra-colonic manifestations can lead to performing endoscopic examination of the large bowel. For example, identifying a desmoid or a mandibular osteoma in an individual should lead to a work-up for ruling-out FAP. This should initially be done by taking a detailed extended family history and performing a sigmoidoscopy or a full colonoscopy depending on the age of the patient or whether we suspect FAP or AFAP.

During childhood, only diminutive adenomas may be found, limited mainly to the rectosigmoid area of the colon, by a *flexible sigmoidoscopy *(Fig. 2A). Random biopsies can visualize intra-mucosal adenomas (Fig. 5D). As age progresses, hundreds of colorectal adenomas and, in some patients, adenomas in extracolonic locations may be found (as described previously) (Figs. 2B and 3A).

The diagnosis of AFAP is more complex than that of classic FAP because of the wide phenotypic variation of disease. Total colonoscopy, rather than sigmoidoscopy, has been advocated for screening individuals at risk as the polyps tend to have a right-sided distribution [25]. Chromoendoscopy (Fig. 5A and 5B) is recommended to highlight the polyp burden. As in FAP, the diagnosis of AFAP is based on the combination of clinical findings and genetic tests.

Genetic testing is mainly used for screening and the presymptomatic early diagnosis of at-risk family members. In addition, confirmation of the diagnosis in patients with obscure clinical findings is essential. At first, only the index case should be tested and because of the long time it can sometime take to receive the answer to the initial mutation analysis, and even if no mutation is identified, first-degree relatives should be managed clinically until results are obtained. If the mutation has been identified, it can be quickly and cheaply performed to screen at-risk relatives.

### Genetic tests in brief

Today, a number of genetic tests are available to test for *APC *germline mutations. Among these are sequencing of the full *APC *gene, combination of conformation strand gel electrophoresis (CSGE) screening and protein truncation test (PTT), protein truncation test alone and finally linkage analysis. The most commonly used today is direct sequencing of the *APC *gene.

The mutation detection rate, when full gene sequencing is performed, is 70%. Large insertions and deletions need other tests and add about 5%. *MUTYH *mutations (see below) are responsible for a subset of the remaining cases.

When the family's specific *APC *mutation is identified, genetic testing of all first degree relatives should be performed even when the parents test negative [28,35,39]. Parents of children at-risk should be advised that genetic testing is recommended just before puberty or preferably in mid-adolescence, when the diagnosis begins to gain clinical importance in terms of cancer prevention *i.e. *surgical intervention, and the child is mature enough to understand the reason for a test and is compliant for evaluation. The managing clinician will occasionally ask for an earlier test if there are suspicious symptoms in the child or extreme anxiety of the parents. Family members who test negative for a known mutation do not need further investigation or follow-up other than standard average-risk screening.

In approximately 20-30% of patients no germline mutation can be found, although the success is improving with extensive testing. In this circumstance, no genetic testing is useful or necessary in any family members; clinical diagnosis and systematic surveillance is mandatory for all first degree relatives.

The genetic alteration in AFAP is associated with *APC *mutations occurring most commonly at the 5' or at the 3' end of the gene proximal to codon 1517 or distal to codon 1900 [39]. Thus, DNA sequencing is usually recommended. The indication for genetic testing include: clinical criteria that meet AFAP or having a first-degree relative with a known *APC *mutation or multiple adenomas.

### Tests for FAP: why the different methods, why the testing is complex and not always complete and why it can be expensive?

The relatively long mRNA makes the screening process for the identification of point mutations by direct sequencing a labor intense and costly method (about 30 PCR fragments are needed to cover all coding exons sequences). Mutation detection in the *APC *gene can be performed using different methodologies. Selection of the methodology depends on the availability of the resources in the laboratory and number of samples analyzed at that time [33].

Several clinical laboratories currently use the RNA based (PTT), also known as the *in vitro*-synthesized protein assay (IVSP), which has a sensitivity ranging from 70% to 90%. However, the disadvantages of the PTT approach include decreased RNA stability in blood lymphocytes, assay artifacts, and an inability to detect non-truncating mutations. Additionally, not all laboratories actually characterize (*i.e.*, sequence) the putative mutation implicated by a PTT alteration.

Less popular methods of heteroduplex analysis include analysis of single-strand conformation polymorphisms (SSCP), conformation-sensitive gel electrophoresis (CSGE), denaturing gradient gel electrophoresis (DGGE) and sequencing the entire coding region of the *APC *gene.

Mutation scanning using denaturing high-performance liquid chromatography (DHPLC) is a highly sensitive method [40], and has been used effectively to screen for mutations in a number of genes [41]. This methodology is useful in a low- to medium-throughput laboratory. When this apparatus is not available, sequencing is the gold standard for mutation detection. Sequencing of the entire coding region of the *APC *gene is only economical in very high-throughput laboratory with a dedicated sequencer and appropriate sequence analysis software.

Approximately 20% of classic FAP patients and 70% to 80% of AFAP cases are negative for an *APC*/MUTYH (see below) point mutation. Therefore, techniques are used for detecting copy number alterations (big deletions or insertions, such as a whole exon or several exons), like using the multiplex ligation-dependent probe amplification (MLPA) method [42]. Real-time fluorescent PCR also allows rapid detection of dosage differences of one versus two copies of the *APC *gene. This methodology permits the detection of gross alterations in the *APC *gene simultaneously with amplification, unlike multiplex PCR or other methodologies such as Southern blotting.

### Genetic differential diagnosis-MUTYH, in brief

A subset of patients that presents with clinical AFAP harbor a recessive disorder caused by the inheritance of mutations in the base-excision-repair gene *MUTYH *[42]. In health, it encodes a protein responsible for the excision of adenosine mismatched with a product of DNA damage caused by reactive oxygen species.

*MUTYH *mutation causes the polyposis condition known as *MUTYH *Attenuated FAP (MAP). It is recessively inherited and patients have either a homozygous or compound heterozygous germline mutations of the *MUTYH *gene. In one series of cases biallelic germ-line *MUTYH *mutations were found in 18% of *APC *gene mutation-negative patients with attenuated phenotype [43].

It is recommended that patients, who have a recessive family history compatible with AFAP, be evaluated for a *MUTYH *mutation. There are reports that patients heterozygous for *MUTYH *may also develop a phenotype of polyposis and because of the high carrier rate, ~1%, there can be compound heterozygotes of uncertain clinical significance [44-46].

### Normal MUTYH structure and functions in humans

**Figure 8:**
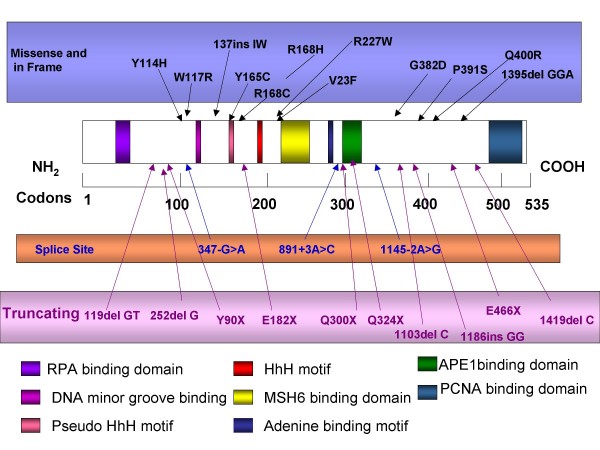
**Diagram of the *MUTYH *protein in scale; known functional domains (the filled boxes) data derived from Sampson et al, 2005 **[43]**; and three types of well known typical mutations - missense or in-frame, splice and truncation**. APE1, apurinic endonuclease 1; HhH, helix-hairpin-helix; PCNA, proliferating-cell nuclear antigen; RPA, replication protein A.

The *MUTYH *gene **- **formerly *MYH*, encodes a DNA glycosylase enzyme which is involved in oxidative DNA damage repair. The enzyme is involved in base excision repair (BER), and excises adenine bases from the DNA backbone at sites where the adenine, after the DNA replications, was inappropriately paired with guanine, cytosine, or 8-oxo-7, 8-dihydroguanine (8-oxoG) which is recognized and catalyzed by the MUTYH protein [47]. The protein is localized to the nucleus and mitochondria [48]. Multiple transcript variants encoding different isoforms have been found for this gene [43]. The *MUTYH *gene contains 16 exons spanning a region of 11147 bp, but only 15 are coding exons [2-16], and it maps to chromosome 1p32-34. The transcribed mRNA is 1854 bp long (NCBI# NM_102222.2) and it encodes a protein with 546 amino acid (NCBI# NP_036354.1) and has a molecular weight of 52 kDa. The protein contains several functional domains including: the N-terminal domain on the 5' side which contains the catalytic region and includes a helix-hairpin-helix (HhH), pseudo HhH and an iron-sulfur cluster loop motif, which are also common motifs in other BER glycosylases; the C-terminal domain on the 3' side shares homology with *MTH*1 (member of the base excision repair (BER) family) and plays a role in 8-oxoG recognition. The *MUTYH *has also binding sites for a DNA binding domain, an adenine binding motif and several interaction domains for apurinic endonuclease 1 (APE1) enzyme, proliferating-cell nuclear antigen (PCNA), replication protein A (RPA) and *MSH*6, located in different regions of the gene (Fig. 8).

### *MUTYH *- disturbed structure and associated polyposis (MAP)

DNA alterations in the *MUTYH *gene at functional domains can alter the proper protein function or abort it completely [49]. For example, one alternative splicing mutation can generate a gene product of 521 amino acids (instead of 546) and is referred to as type 2. Type 1 is transported to the mitochondria, while type 2 lacks the first exon containing a mitochondrial targeting signal (MTS) and is transported to the nucleus.

Mutations in this gene result in a heritable predisposition for colon and occasionally stomach cancers [49]. *MUTYH *associated polyposis is mostly an autosomal recessive disorder, the frequency of heterozygotes carriers is 1-2% and the frequency of bi-allelic mutation carriers lies between 1 per 10,000 and 40,000 births. The penetrance for colon polyps is close to 100% and bi-allelic *MUTYH *mutation carriers generally develop 10-100 adenomatous polyps/adenomas of the colon and rectum [49]. In some heterozygous *MUTYH *mutation carriers (apparently a dominant form of MAP), a slightly increased risk for developing CRC has been found [45,46]. Approximately one third of patients also develop polyps/adenomas in the upper gastrointestinal tract [50].

Because of the development of multiple polyps, the risk for CRC is high and 60-70% of MAP CRC patients were first diagnosed at a mean age of 47 years. When frequent colorectal and upper gastrointestinal tract screening is performed in a MAP-patient who has not developed cancer, the chance of developing carcinoma is small and prognosis will be comparable to that of a healthy population.

### *MUTYH*-genotype/phenotype correlations to "FAP" features

A large proportion of non-FAP non-Lynch syndrome patients with multiple colorectal adenomas have germline mutations on the *MUTYH *gene. Although the number of adenomas appears to be dependent on the number of mutated *MUTYH *alleles present in a patient, little is known on the relation of this number with cancer risk. This recently recognized type of adenomatous polyposis was first documented in a Welsh family having bi-allelic germline mutations [51]. Similar findings were subsequently confirmed in other subjects of European origin having multiple colorectal adenomas ranging from 5 to several hundreds [52]. There are some ethnic specific mutations. While the mutations Y165C and G382D are the most common in Europeans, the Y90X mutation occurs in subjects of Pakistani origin, whereas an E466X mutation has been found in subjects of Indian origin [43]. An in-frame deletion nt1395-7delGGA has been described in Italian populations [53]. Baglioni *et al. *2005 identified in a brother and sister, the offspring of first-cousin parents, an association of multiple adenomatous polyps of the colon with childhood pilomatricomas, a homozygous 2-bp insertion in exon 13 of the *MUTYH *gene, 1186insGG, resulting in a frame shift and a premature stop codon at position 438 [53]. The brother also had early-onset rectal adenocarcinoma. In a large screening of 453 *APC*-negative patients with more than five colorectal adenomas, it was found that pathogenic mutations were initially found in 74 patients without extra-digestive tumors (22.5%) and subsequently in 75 at-risk relatives [46]. Polyposis was more severe in cases with bi-allelic mutations. However, mutation copy number was correlated neither with the age at diagnosis of adenomas or adenocarcinomas, nor with the presence of a family history of colorectal tumors. Heterozygous and homozygous *MUTYH *mutation carriers were both at high risk for synchronous cancers (24% in the colorectum and 16% in the upper gastrointestinal tract), but did not demonstrate an increased risk for extra-digestive tumors [46]. Recently, it has been demonstrated that *MUTYH *polyps may not only be adenomas, but also sessile serrated adenomas and hyperplastic polyps [54]. These do not exclude the diagnosis of a *MUTYH *mutation.

### Tests for MUTYH

Genetic testing for *MUTYH *mutation has been recommended for all patients who have tens to hundreds of colorectal adenomas with no identified germline mutation in the *APC *gene and with a family history compatible with an autosomal recessive mode of inheritance. *MUTYH *associated polyposis is a frequent inherited CRC predisposition, which can be mostly in a recessive form of inheritance (bi-allelic or compound mutations) but also as a dominant component, and therefore DNA screening of the *MUTYH *gene should look for both heterozygous and homozygous mutations [46]. Mutations screening can be done by direct sequencing of the entire coding region of the *MUTYH *gene, but this is time-consuming and costly (sequencing 15-16 PCR fragments in a bidirectional way). DNA chromatography (DHPLC) as first line of screening could be applied by subjecting PCR products to chromatography using an ion-pair reversed-phase cartridge. PCR products are denatured and allowed to re-anneal. Under conditions of partial denaturation with a linear acetonitrile gradient, heteroduplexes from PCR samples having an internal sequence variation display a reduced column retention time relative to their homoduplex counterparts. The elution profile for heterozygous samples is typically quite distinct from that of either homozygous sequence, making the identification of heterozygous mutations relatively straightforward [42]. An analysis for homozygous autosomal mutations requires mixing the test sample with the DNA of a known sequence [55]. Approximately 20% of classic FAP cases and 70% to 80% of AFAP patients are negative for the *APC/MUTYH *point mutation. Therefore, techniques for detecting copy number alterations should use MLPA [56].

### What to test for first: APC or MUTYH mutation?

Because the carrier mutation rate in the *MUTYH *gene, is about 1% of patients with low or mild polyposis and it has been found that heterozygous *MUTYH *mutation carriers can have an increased risk for developing CRC (especially in MAP patients aged >55 years), this can be a dilemma. If the patient's phenotype and family history are compatible with FAP, one should start by screening the *APC*, as this gene is more likely to be mutated then the *MUTYH*.

One can combine testing for the two genes when planning a strategy for screening both in polyposis patients. Screen the sequences of the *APC *exons 2-15 and 2,500 bp of the 5' of exon-16 together with the 15 coding exons of the *MUTYH *gene and only when no mutations are found then to continue to screen the rest of the *APC *(exon-16, the 3' end which has 4076 bp and infrequent mutations). DNA chromatography can be used to reduce the time and cost for this type of screening. If no mutations are detected in both genes, one can continue to do MLPA, but this method is relatively expensive.

If the mutations that were found in one of these *MUTYH *genes are heterozygous mutations, only then should the second gene be screened, as one heterozygous mutation does not usually fully explain the different phenotypes (number of polyps, colonic distribution, age of onset, family history). Two severe mutations in both genes might have a cumulative effect and a strong clinical impact that could lead to illness at a young age and the phenotype will be more severe. We estimate that a mutation in the *APC *gene will be more severe then mutation in the *MUTYH *gene, as the *APC *gene is part of the Wnt pathway and can influence several cellular functions such as proliferation, differentiation, apoptosis, adhesion, migration, and chromosomal segregation. While *MUTYH *is an enzyme that fixes damage in the DNA after replications, and more then 90% of DNA is not a coding or regulatory element of the genome, so most of the DNA replication mistakes, will not have a prominent effect.

## Differential diagnosis of FAP

There are other disorders causing multiple polyps. These include hamartomatous polyps such as those in Peutz-Jeghers syndrome (mainly in the small bowel but may occur anywhere along the gastrointestinal tract), familial juvenile polyps or hyperplastic polyposis and hereditary mixed polyposis syndromes. Multiple lymphoid aggregates can masquerade as early FAP, especially in children and young adults. The diagnosis depends on the correct histological classification of the polyps. Dysplastic changes occurring in a non-adenomatous polyp can be mistakenly identified as a multiple adenoma syndrome compatible with FAP. The combination of adenomatous polyps and an autosomal dominant pattern of inheritance is classic for FAP and rules out most of the alternative possibilities. At times it is difficult to differentiate between AFAP and Lynch syndrome (hereditary non polyposis colorectal cancer as both may have a low polyp burden, occurring mainly in the right colon. The differentiation from MAP is discussed above.

## Genetic counseling and socio-legal aspects of FAP

FAP is a hereditary condition, so referral to a geneticist or genetic counselor is mandatory [1,57]. FAP is a dominant syndrome caused when one copy of the *APC *gene contains a fault; this means that every child of a FAP patient has a 50% chance of inheriting the faulty gene. There is a risk of FAP in first-degree family members who may also be symptomatic or even asymptomatic at the time of diagnosis in the propositus. The risk to the siblings depends on the genetic status of the parents who need to be evaluated for carrying the mutation. As mentioned above, because of germline mosaicism, this does not exclude siblings being affected and they should also be evaluated genetically [23]. About 20-30% of probands have a *de novo *mutation.

The diagnosis of FAP, with or without an identified genetic mutation can lead to guilt, anxiety, cancer phobia, denial and refusal to collaborate. These patients and their families need a treating physician who understands the complexity of their condition and can provide clear advice. Affected children, who have not been adequately counseled, may develop antagonistic feelings towards the disease-transmitting parent and they may need help from a child psychologist who understands the medical problem, especially if they need to undergo a potentially traumatic or mutilating procedure such as creation of a stoma. As many of the patients may be underage, informed consent from the parents must be obtained for all genetic testing and other invasive procedures. Whenever possible the child should also be given an explanation and understand why the testing is being performed [58]. So, a geneticist or genetic counselor is needed prior to genetic testing, and is also important when dealing with prenuptial young adults. Not involving the future spouse can lead to extreme strains on intra-familial relationships when the medical condition and risk for FAP and, or, cancer in the patient and children becomes known.

The genetic information is not only relevant to the affected patient, but also to the immediate family and even to future descendants. This leads to ethical dilemmas and patients, for personal or religious reasons, may even refuse to allow the information to be provided to the unsuspecting immediate family at risk [59,60]. Failure to obtain a relevant family history and instructing the family on their risk and need for follow-up can be considered medical negligence [61]. However, it is not clear how this can be done without the patient's consent. Another common problem is that by identifying an asymptomatic person at-risk and needing diagnosis and follow-up could lead to losing a job and medical and life insurances [62,63]. These fears inhibit the patient's cooperation and these problems need to be addressed by legislation.

## Prenatal diagnosis, pregnancy and genetic counseling

Identification of the mutated *APC *gene responsible for FAP enables presymptomatic and even prenatal diagnosis of the disease. If a mutation is identified in a family member then prenatal testing can be performed. Conventional prenatal diagnosis such as *amniocentesis *and *chorionic villous sampling *(CVS) could indicate whether a fetus is affected, giving the option to selectively terminate the pregnancy. The question that arises is how acceptable is prenatal diagnosis and pregnancy termination among FAP kindred? The answer to this complex question will be influenced by experience with the disease, psychological as well as cultural and religious attitudes. In the UK, 62 adults were questioned regarding antenatal testing and only 24% of those questioned stated that they would proceed to termination of pregnancy if a prenatal test indicated that the unborn baby was affected; however, in clinical practice this was not requested [64]. A study that included 20 FAP patients found that 90% would consider preimplantation genetic diagnosis, 75% would consider amniocentesis or CVS. Having an affected child and experiencing a first-degree relative's death due to FAP were associated with greater willingness to consider prenatal testing [65]. Another study [66], that included younger individuals and individuals "at risk" for FAP, found that although 75% would consider prenatal genetic testing only 21% would consider termination of an affected pregnancy. The predictive testing of a fetus for a disease that has an affect on quality of life, as well as early mortality, should be offered to couples who would be willing to avoid the birth of an affected child or terminate a pregnancy at an early stage.

Amniocentesis, the test that is most commonly used, is performed between 16 and 20 wk of gestation. A small-bore needle is inserted through the abdomen and uterus, into the amniotic sac, usually with ultrasound guidance, and approximately 30 mL of amniotic fluid is drawn for analysis. CVS allows for earlier test results and is performed between 10 and 12 wk. CVS is usually performed trans-cervically, with ultrasound-guidance, a thin plastic tube is inserted into the placenta and a small sample of chorionic villous tissue is withdrawn by suction. The risk of miscarriage estimated to be between 0.25-0.5% for amniocentesis and 0.5-1.0% for CVS [67].

An additional alternative to prenatal diagnosis and termination of pregnancy is *preimplantation genetic diagnosis *(PGD). This method enables genetically affected individuals to produce healthy fetuses, by selection of embryos that are free of the genetic mutation that leads to FAP. By ensuring unaffected pregnancies, PGD avoids early pregnancy termination with all that is involved. PGD, even if subject to controversy, expensive and not easy to perform (both for the patient and laboratory), seems to be a more acceptable option than prenatal diagnosis.

Two methods for PGD have been recently developed and implemented in the framework of *in-vitro *fertilization. PGD can be performed by micromanipulation and biopsy of the first polar body before fertilization, or by blastomeric biopsy before implantation of the pre-embryo. Available data suggest that preimplantation diagnosis is safe, as no detrimental effects have been observed in studies on the viability of biopsied pre-embryos. Genetic analysis of biopsied gametes and blastomeres is now possible by DNA analysis, while enzyme analysis and preimplantation diagnosis of chromosomal disorders are still at the research stage. The accuracy of DNA analysis in preimplantation diagnosis is clear from available data on the outcome of these pregnancies. To date, children free of genetic diseases have been born following preimplantation diagnosis of cystic fibrosis, hemophilia A, FAP, and other conditions. In individuals with FAP, Moutou *et al. *[68] reported 11 cycles that were performed for four couples, resulting in eight embryo transfers and five pregnancies, with the birth of one healthy boy and two ongoing pregnancies. In Australia, Davis *et al. *in 2006 reported performance of PGD. After standard IVF hormonal treatment 14 oocytes were collected, 11 inseminated and nine embryos were biopsied on day 3. Of the nine embryos that were analyzed, five embryos were affected and four were unaffected. Two unaffected embryos were transferred on day 4 resulting in a triplet pregnancy and the birth of three healthy babies [69]. Today, PGD is an acceptable and potentially preferable method to reduce the birth risk of babies affected with FAP.

### Pregnancy

During pregnancy, due to the endogenous multiple growth factors and hormones, there is an increase rate of desmoid and adenoma development in the mother. However, if possible, therapies should be delayed until pregnancy has ended so as to avoid the possibility of fetal damage.

## Management of the FAP patient

Cancer prevention and maintaining good quality of life are the main goals in management of patients with clinical or genetic evidence of FAP. Large bowel endoscopy is the most important clinical examination since there is almost a 100% chance of CRC. However, as discussed previously, the disease is systemic with extracolonic manifestations and should be looked for by systematic reexaminations [11,70,71]. CRC is rare in the asymptomatic youth, so after their genetic diagnosis and baseline sigmoidoscopy, they are systematically followed clinically until completing schooling and growth and maturing. Around ages 16-18 y patients with FAP should be followed by annual or less frequent colonoscopic examinations (depending on the polyp burden at last colonoscopy) and all significant sized adenomas should be removed if surgery is not contemplated at that time. In addition, both forward-viewing and side-viewing upper tract endoscopies should be performed prior to surgery or every 1 to 5 years depending on the polyp burden and Spigelman stage (Tables 1 and 2) [8,72] to detect gastric but mainly duodenal and periampullary adenomas, respectively.

**Table 2:** Recommended surveillance interval intervals between gastroscopy examinations

Spigelman 0 and I:	Endoscopy at intervals of 5 years
Spigelman Stage II:	Endoscopy at intervals of 3 years
Spigelman Stage III:	Endoscopy at intervals of 1-2 year
	Consider endoscopic ultrasonography Consider celecoxib 800 mg/d
Spigelman Stage IV:	Endoscopic ultrasonography (EUS)
	Consider surgery: Pancreas-sparing or pylorus-sparing duodenectomy

Source, reference [12]

Usually, by the late teens or early twenties, due to the increasing number of adenomas, *prophylactic cancer-preventive colorectal surgery *is advocated [27,72-74]. Since this is an elective procedure the timing can be arranged to be the least inconvenient for the patient and family. Elective surgery, can at times, be delayed if the patient is compliant and polyps are sparse and not large.

Surgical options include subtotal colectomy with *ileorectal anastomosis *(IRA), total proctocolectomy with *ileostomy*, and proctocolectomy with or without mucosectomy and *ileal pouch anal anastomosis *(IPAA) [75-77].

Given the substantial risk of rectal cancer developing after colectomy and ileorectal anastomosis, most experts advise total proctocolectomy for the typical FAP patient with multiple rectal adenomas [77-79]. This surgery includes removal of the entire large bowel and striping of the remaining rectal mucosa down to the dentate line if there are multiple polyps or leaving a cuff of rectal mucosa, and forming an internal pouch from the ileum. Because of better bowel control after such surgery, many colorectal surgeons prefer the stapled rather than the hand-sewn anastomosis. So, if there is no severe rectal polyposis this is the most common pouch operation. However, this requires careful biannual or annual examination and removal of adenomas that can recur there [80]. This surgery is called total proctocolectomy with ileoanal J-pouch and it is the surgical procedure of choice for most patients with classical FAP [79] (Figs. 3B and 3C).

In cases with few rectal polyps, IRA can be a suitable alternative procedure providing there is acceptance of life-long rectal surveillance [75]. Many prefer this procedure for women with a low polyp burden since it has been reported that pouch procedures can reduce fertility [81,82]. Later conversion of an ileorectal anastomosis to a J-pouch can be performed, but may be difficult because of desmoid formation in the operated area.

### Post-colectomy surveillance

**Figure 9:**
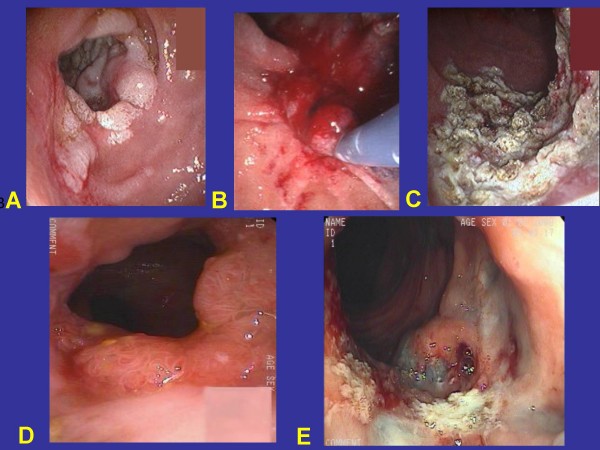
**Panel A shows flat and elevated duodenal adenomas in a FAP patient**. These were proven by biopsy and endoscopic ultrasound to be free of cancer. Panel B - the polyps were removed by submucosal resection and polypectomy. Panel C - the polyp remnants had been destroyed by argon plasma coagulation (figures provided by Dr. M. Santo, Tel Aviv). Panel D shows a large polyp that developed, during pregnancy, in the ileo-anal pouch of a FAP patient. Panel E - the polyps had been removed by submucosal resection and polypectomy and remnants destroyed by argon plasma coagulation (figures provided by Dr. Z. Halpern, Tel Aviv).

It is important to emphasize that follow-up is vital after surgical procedures are completed. Initially, it should be at short intervals to asses the psychological and physical adaptation to surgery and identify desmoid tumor formation in its earliest stage. The initial follow-up should include a thorough physical examination, baseline abdominal ultrasound (US) or computed tomography or magnetic resonance imaging (CT or MRI) to aid in detecting existing or future changes suspicious of a desmoid tumor. Patients after stapled and hand sewn IRA are at risk for rectal adenomas and carcinomas. Therefore, the physician needs to stress the importance of endoscopic annual surveillance of the pouch. Many studies have shown that adenomas and occasionally even adenocarcinomas have been found in the ileo-anal pouch after restorative proctocolectomy (Figs. 9D and 9E). Therefore, surveillance of the pouch [80,81,83] and transitional anal zone [84] is essential.

### Quality of life after surgery

Since surgery is an elective procedure in FAP, the treating specialist has the opportunity to educate the patient regarding the specific procedure and quality of life that should be expected post-surgery so as to minimize fear and reduce expectations. This can be facilitated by meeting patients of the same sex and similar age group who have had a similar procedure performed, and occasionally a sympathetic psychologist can be helpful in overcoming fear [70,85]. There are many reports showing that most patients are satisfied following an IPAA procedure [82,86,87]. However, patients should be advised that although fecal elimination via the anus will be preserved, functional outcome may vary and is not comparable with bowel elimination prior to surgery. Pouchitis is a major cause of morbidity and discomfort in patients undergoing IPAA for ulcerative colitis (15-20%), however, this is rare in FAP patients (0-10%).

The majority of patients with FAP develops adenomatous polyps and requires preventive surgery during their late teens or early twenties. These years are the main reproductive years and maintaining sexual function is of major concern. Sexual impairment following proctectomy is largely technique-dependent and this should be discussed with the surgeon performing the procedure [82]. For men, denervation of the pelvic plexus is the major cause of erectile and ejaculation dysfunction. Following an IPAA, erectile dysfunction is reported to occur in 0-1.5% of patients, while ejaculation dysfunction occurs in 3-4% of these individuals [86-89].

In woman, sexual dysfunction is less obviously disturbed, mostly due to the fact that it is more difficult to measure. In addition, there is a lack of reporting of discomfort as well as dysfunction. Dyspareunia is a major concern and in different reports affects between 3-22% of the patients [81,88,89]. This may be due to anatomical changes within the pelvis following proctectomy. Approximately 3% of woman report avoidance of sexual contact due to fear of fecal leakage. For these reasons surgical experience with the IPAA procedure is of great importance and patients with FAP are advised to have the procedure performed in medical centers that are familiar with FAP and by surgeons experienced with this procedure. Knowledge about fertility of women suffering from FAP is scarce and inconclusive. The IPAA procedure does not risk pregnancy but may reduce fertility. Olsen in 2003 [82] reported that fecundity dropped to 54% following proctocolectomy with IPAA, a rate similar to that in patients undergoing IPAA for other indications although it was greater than the postoperative fecundity of women with ulcerative colitis. It is thought that pelvic adhesions after surgery may be responsible for infertility in FAP woman post IPAA. The significant reduction in female fecundity after IPAA should be discussed with women with FAP before it is decided which surgical option to choose and timing of the operation. If the mild manifestation of FAP make it possible, and the patient is compliant for frequent follow-up, then elective surgery should be delayed until completing the planned family.

## Extra-colonic disease

*Adenomatous polyps *are also found in the stomach and duodenum, especially the *periampullary area *and can develop into adenocarcinomas. After colectomy, periampullary carcinoma is the most common malignancy, occurring in approximately 5-6% of the patients. It is the major cause of death in patients with FAP who have had prophylactic colectomy. For this reason upper endoscopy with a gastroscope and a side-viewing duodenoscope should be performed every 1-5 yrs depending on the gastric and specifically duodenal and periampullary polyp burden. The EUROFAP guidelines for surveillance are presented in Table 2. After adequate biopsy sampling of the lesions to determine the degree of adenoma dysplasia and endoscopic ultrasound to determine depth of mucosal involvement, large polyps can be treated with endoscopic mucosal resection. Argon plasma coagulation is used to destroy small adenomas and polyp remnants after mucosectomy, but when repeatedly performed it causes scaring and duodenal narrowing which may require dilation (Figs. 9A-C). Endoscopic ampulectomy can be performed, but with stenting of the pancreatic duct so as to minimize post-procedure pancreatitis. There is theoretical, but unproven evidence that changing the cancer-promoting bile to ursodeoxycholic acid by saturating it with ursodiol could be useful. This could be combined with celecoxib.

Documentation of villous changes, severe dysplasia, and rapid growth of an adenomatous polyp has also been suggested as indications for surgical intervention. Spigelman stage 4 is an additional indication for surgery. The procedure of choice for many years has been the standard Whipple procedure. Pylorus preserving duodenectomy, performed in centers where physicians are familiar and experienced with this procedure, or pancreas preserving duodenectomy (PPTD) which includes resection of the entire duodenum from the pylorus to the ligament of Treitz with preservation of the pancreas have both been successfully performed [90,91]. PPTD is a safe surgical procedure for duodenal adenomatosis, provides high quality of life, and shows advantages over the pylorus preserving - Whipple procedure [91].

*Desmoid tumors *occur in approximately 10% of patients with FAP, more often in women, and are known to be induced by surgical procedures and pregnancy. They are major causes of morbidity by compression of organs or vessels such as ureter or blood supply, can bleed into the gastrointestinal tract, or if not managed early can cause death by massive bowel compression. Most of these tumors have ill-defined borders, are located in the mesentery, areas of scars or bowel wall, but can also be detected in many other locations. Any suspicious finding on physical examination requires imaging studies [92]. Treatment of desmoid tumors should be under supervision of a multidisciplinary team that includes surgical and medical oncologists, and gastroenterologists who are familiar with the disease. Treatment is first aimed at measuring the individual's risk from the disease itself and potential benefit of intervention. Pre-treatment staging based on tumor location; size, symptoms and growth behavior are most useful [93]. In most cases, a period of initial observation to see if its size is static, regresses or progresses, is an option. There are no controlled trials of therapies, but there are small and positive experiences reported with anti-estrogens (*e.g. *tamoxifen) [94] and COX-2 inhibitors (sulindac or celecoxib) in chemoprevention and slowing progression of the disease; radiation has also shown some effectiveness [95]. Surgery is performed when necessary, often for abdominal wall desmoids. Usually, the procedure cannot completely remove the mass and may actually stimulate re-growth there or elsewhere. Desmoids bleed on biopsy (which should be avoided) and surgery, and this is difficult to control. For tumors managed by surgery the objective is to achieve tumor free margins (R0) without sacrificing small bowel. These therapies are most useful when the lesions are small. Multiple, small case-series report objective responses to cytotoxic chemotherapy in severe desmoid disease. These include doxorubicin and dacarbazine, followed by carboplatin and dacarbazine, the vinca alkaloids vincristine, vinblastine and vinorelbine, and the combination of vinblastine with methotrexate. In a relatively large series of 21 patients with FAP (Bertagnollia *et al. *[96]), 93% of patients with progressing desmoid tumors and unresectable disease achieved disease response or stability with the combination of cytotoxic chemotherapy that included liposomal doxorubicin as first line therapy and vinorelbine used as second line and surgery when possible.

To summarize, the optimal management strategy for desmoid tumors in patients with FAP should be individualized, taking into consideration the extent of disease, morbidity and potential benefit versus risk of the different treatment modalities.

*Osteomas *are common and are usually left alone unless they are unsightly or interfering with the patient's function. Therapy is initially as for desmoids, but they might require reconstructive plastic surgery if disfiguring.

*Small bowel adenomas *occur and are rarely a problem to the patient, although cancers have been reported [97]. The cancer can occur in adenomas close to the duodenal bile-flow (as discussed earlier) or close to the ileo-anal anastomosis. The role of wireless *capsule endoscopy *(CE) in surveillance of the asymptomatic patients with FAP is presently unproven. A small study of 23 individuals with FAP [15] found 11 patients with duodenal polyps and 7 had jejunal-ileal polyps. CE missed ampulary and many of the duodenal polyps detected at endoscopy, but were successful in identifying mid-distal small bowel polyps. Burke *et al. *[16] reported that 9 of 15 (60%) of subjects with FAP had small bowel polyps. The prevalence of small bowel polyps was related to the duodenal polyposis stage and subject's age. The location, size and number of polyps progressed as duodenal polyposis stage advanced [16]. On the other hand, Wong and colleagues [98] from Utah found that CE underestimated the number of small bowel polyps and did not reliably detect large polyps. Additional studies are required before recommendations can be made.

*Other extra-gastrointestinal tumors *are rare and have been reported as pancreatic adenocarcinoma (2%), thyroid cancer (2%), gastric adenocarcinoma (0.5%), and hepatoblastoma in children less than 5 years of age (1.6%). These should be searched for during routine clinical and imaging follow-up.

There are non-somatic perturbations related to the diagnosis and therapy of FAP (see section on Genetic counseling).

## Effectiveness of screening

The usefulness of screening asymptomatic FAP patients for all of its possible manifestations is unproven. For children, to identify hepatoblastoma, some recommend annual alpha-fetoprotein and abdominal ultrasound from birth until the age of 10 years. For all FAP patients, an annual physical examination should include an evaluation for soft tissue or bone lesions, and a thorough thyroid examination with a low threshold for performing an ultrasound of any suspicious lesion.

Symptomatic patients (abdominal pain, new onset diabetes mellitus or acute pancreatitis) require evaluation which could include computed tomography of the abdomen to rule out desmoid tumors of the mesentery or pancreatic adenocarcinomas (or intraductal papillary and mucinous tumors (IPMT) of the pancreas [99]. If the CT is not diagnostic, then magnetic resonance imaging is indicated, it can outline vascular involvement of a desmoid tumor and may predict its growth [100]. CT of the brain can also be used in symptomatic patients to search for a medulloblastoma.

Individuals with a *family history *of FAP should be screened. When a specific *APC *mutation has been identified in an index patient all 1^st ^degree relatives carry a 50% risk of FAP and should be referred for genetic counseling and offered *APC *mutation testing. A family member who is found to carry the mutation has 100% chance of developing FAP and its complications. It is recommended that these individuals have a colonoscopy and follow specific surveillance recommendation that have been outlined for FAP patients. Children that have been identified as carrying an *APC *mutation should have a flexible sigmoidoscopy performed by the age of early adolescence unless symptoms develop at an earlier stage. When polyps are detected, discussion with the patient and parents should take place regarding further surveillance and timing of surgery as described above.

If genetic testing cannot be performed in a 1^st ^degree relative of a known mutation carrier, then the asymptomatic 1^st ^degree family member has a 50% chance of harboring the mutation and should be screened as if they have the *APC *mutation. All adults should have colonoscopies performed and all children should undergo a flexible sigmoidoscopy around the age of 10-12 years [101]. Adenomas develop with the child's growth, and therefore are easier to identify at adolescence. If polyps are not found, then there should be annual clinical visits for physical or ophthalmic evidence of FAP and to assess suspicious symptoms. Sigmoidoscopy should be repeated at suitable intervals, minimizing its psychological trauma and maximizing cooperation of the growing child, until polyps emerge. If by the age of 25 years polyps are not detected, then biennial sigmoidoscopy or, preferably colonoscopy, can be done since the likelihood of developing adenomas decreases as the patient's age increases. From the age of 35 years, an examination every third year is recommended until the individual is 50 years old. If polyps have not been detected by then, the individual most likely, but not absolutely, doesn't have FAP, and screening is recommended according to guidelines for the general population.

When an individual in a family with a known mutation tests negative, then routine colorectal screening is recommended, as for the general population, beginning at age 50 years.

In approximately 25-30% of patients with clinically evident FAP a mutation cannot be identified. If available, *MUTYH *testing and more complete DNA analyses are performed in specialized laboratories. If these are also negative, then such individuals are considered to have FAP and should be treated as such. Gene testing can exclude FAP only if a mutation is identified in a family member and this mutation does not exist in a given individual.

## Prevention

### Diet and lifestyle

The evidence for being able to modulate the clinical manifestations of a dominant genetic disease is indirect and based on observations in animal models and humans. Caloric restriction or diet with olive oil, fruits and vegetables significantly reduced the number of polyps in the genetically manipulated *APC *Min mice model [102]. In the same model, low dosage ursodiol together with sulindac prevented adenomas with less toxicity than if each had been given alone in full dosage [103].

Clinical observation of FAP families showed that the severity of disease varied between affected family members or between families carrying the same mutation [104]. This would suggest that not only are there genetic-genetic/endogenous modulating factors, but there could be genetic-exogenous modulating factors at play. Adenoma expression and growth occurs with aging, effect of growth factors, hormones, weight gain, diet, tobacco use. As an example, in two twin boys with FAP there was a clear correlation between obesity in one twin and adenoma expression as compared to the other twin (Rozen, personnel communication).

So, it would be wise to recommend a "balanced" CRC-preventive lifestyle and diet from childhood in anticipation of modulating the clinical expression of FAP.

### Chemoprevention

**Figure 10:**
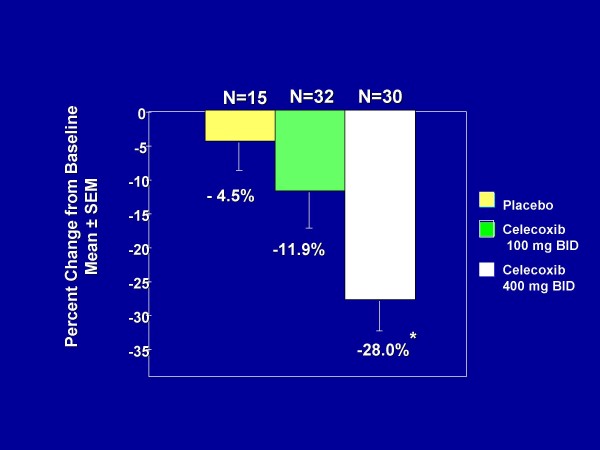
**Change in number of colorectal polyps in FAP patients receiving placebo or celecoxib (Celebrex^R^) for 6 months**. The reduction in polyp burden, versus placebo, was significant (*P = 0.003) with the dosage of 400 mg twice a day (data for figure derived from reference [107]).

Randomized trials have shown that both sulindac [105,106] and celecoxib [107] cause regression of established adenomatous polyps in individuals with FAP. Specific cycloxygenase-2 (COX-2) inhibitors, celecoxib and rofecoxib [108,109], among others, were developed to overcome the risk of gastrointestinal damage due to inhibiting the cytoprotective COX-1. In patients with FAP, treatment with 400 mg of celecoxib twice daily for 6 mo had been shown to reduce the tumor burden by 28% as compared to a reduction of 4.5% in the placebo group (p = 0.003) [107] (Fig. 10). In 2001, the US Food and Drug Administration approved the use of celecoxib (as an adjunct to endoscopic surveillance, and surgical management) in patients with FAP and having polyps; while the European Medicines Agency approved an orphan designation for celecoxib [3]. This agency has also designated eflornithine hydrochloride, an irreversible inhibitor of ornithine decarboxilase (ODC), the first and rate-limiting enzyme in the polyamine synthesis, as an orphan medical product to be investigated for use in individuals with FAP [110].

A recent study failed to show that sulindac prevented the primary development of adenomas in individuals with FAP [111]. However, although relatively safe in terms of gastrointestinal toxicity, the long-term use of COX-2 inhibitors in the setting of CRC prevention carries a risk, albeit small, of serious cardiovascular complications [112]. Celecoxib/sulindac given for adenomatous polyps in the retained rectum or duodenum in order to delay definitive surgery is expensive and intramucosal adenomas and CRC still occurred [105]. This could be due to the untreated COX-1 found in all adenomas [113]. Adenoma regression has occurred also with estrogen/progesterone oral contraceptives [114].

### Attenuated FAP

The management of patients with AFAP depends largely on the polyp burden and their location in the large bowel. In a patient with few adenomas that can all be removed, colonoscopic polypectomy is sufficient. Since the adenoma-carcinoma sequence in these patients does not seem to be overly accelerated, a 2 year interval between colonoscopies, probably for life, could be sufficient. If multiple polyps or clusters are found during colonoscopy, or repeated total colonoscopy is technically difficult, surgical resection is the treatment of choice for these patients. Subtotal colectomy with an ileorectal anastomosis can usually be performed due to the relative sparing of the rectum in AFAP. Systematic rectal surveillance is mandatory after this procedure.

### Management of extracolonic adenomas

This is recommended as outlined for FAP, but the risk of malignancy is much lower.

## Prognosis

The goal is pre-symptomatic genetic diagnosis of *APC *mutation-carriers that can lead to improved clinical care and prevent premature mortality from cancer or other FAP complications. Most patients with clinical FAP can be identified and have their diagnosis confirmed by genetic testing. Individuals with FAP carry a 100% risk of colorectal cancer that is reduced almost absolutely when patients enter a screening-treatment program as outlined earlier. Once proctocolectomy has been performed, the risk of ampullary and duodenal cancer is significant and requires lifelong upper gastrointestinal surveillance that has been shown to save lives of FAP patients. Desmoids need to be identified early while small and not causing local perturbations. They should be managed as described above. Duodenal cancer and desmoids are the two main causes of mortality after total colectomy has removed the risk for CRC. The sociological, psychological and physiological issues related to the diagnosis and treatment of FAP need to be addressed. The colectomy and ensuing change in bowel habits, frequently lead to dietary changes that can be unbalanced and lead to vitamin-mineral deficiencies. Notable is the possibility of vitamin B_12 _deficiency due to rapid intestinal transit, ileal resection and ascending bacterial overgrowth. All these problems require systematic follow-up and supportive care.

## Unresolved questions

There are two major issues that need to be addressed. Firstly, there is a significant minority of patients with clinical FAP that don't have their genetic mutation identified even with the more sophisticated genetic testing. This issue means that first-degree relatives cannot be screened genetically and will require life-time repeated clinical evaluation to exclude being a carrier of the disorder. Secondly, there is some clinical evidence supporting the possibility of influencing the manifestation of disease by chemoprevention and lifestyle changes. These issues require further research and evaluation.

## Abbreviations

AFAP: Attenuated familial adenomatous polyposis; APC: adenomatous polyposis gene; APE1: apurinic endonuclease 1; BER: base excision repair; CE: capsule endoscopy; CHRPE: congenital hypertrophy of the retinal pigment epithelium; CIN: chromosomal instability; CRC: colorectal cancer; CSGE: conformation strand gel electrophoresis; CT/MRI: computed tomography, magnetic resonance imaging; CVS: chorionic villous sampling; DGGE: denaturing gradient gel electrophoresis; DHPLC: denaturing high-performance liquid chromatography; EMEA: the European Medicines Agency; FAP: familial adenomatous polyposis; FGP: fundic gland polyps; GI: gastrointestinal; GSK3: glycogen synthase kinase 3; HNPCC: hereditary non-polyposis colon cancer; IPAA: ileal pouch anal anastomosis; IPMT: intraductal papillary and mucinous tumors; IRA: ileorectal anastomosis; IVSP: in vitro-synthesized protein assay; MAP: MUTYH attenuated FAP; MCR: mutation cluster region; MLPA: multiplex ligation-dependent probe amplification; MTS: mitochondrial targeting signal; ODC: ornithine decarboxylase; PCNA: proliferating-cell nuclear antigen; PGD: preimplantation genetic diagnosis; PPTD: pancreas preserving duodenectomy; PTT: protein truncation test; RPA: replication protein A; SSCP: single-strand conformation polymorphisms; 8-oxoG - 8-oxo-7, 8-dihydroguanine.

## Competing interests

The authors declare that they have no competing interests.

## Authors' contributions

EH wrote the review and was responsible for the contents of the review except for the detailed sections on genes and their testing.

DB wrote the detailed sections on genes, their testing and provided the associated figures.

PR wrote specific clinical sections of the text, provided the figures other than those associated with the genes, and performed the editing.

All authors read and approved the final manuscript.
